# Prevalence and Associated Factors of Self-rated Oral Health among a National Population-based Sample of Adults in Sudan: Results of the 2016 STEPS Survey

**DOI:** 10.3290/j.ohpd.b1749751

**Published:** 2021-07-15

**Authors:** Supa Pengpid, Karl Peltzer

**Affiliations:** a Professor, ASEAN Institute for Health Development, Mahidol University, Salaya, Phutthamonthon, Nakhon Pathom, Thailand; Department of Research Administration and Development, University of Limpopo, Polokwane, South Africa.*; b Professor, Department of Psychology, University of the Free State, Bloemfontein, South Africa; Department of Psychology, College of Medical and Health Science, Asia University, Taichung, Taiwan.*; * Both authors conceived and designed the research, performed statistical analysis, drafted the manuscript, and made critical revisions of the manuscript for key intellectual content. Both authors read and approved the final version of the manuscript and have agreed to the order of authorship for this manuscript.

**Keywords:** adults, health variables, oral conditions, self-rated oral health, Sudan

## Abstract

**Purpose:**

To estimate the prevalence and correlates of self-rated oral health (SROH) among adults in a national population-based survey in Sudan.

**Materials and Methods:**

Nationally representative data were analysed from the cross-sectional 2016 Sudan STEPS survey. In all, 7722 18- to 69-year-old individuals (median age 31 years) were assessed with questions on SROH, physical measurements, and medical conditions.

**Results:**

The prevalence of poor SROH was 8.0%, with 12.4% among females and 4.4% among males. In multivariable logistic regression analysis, ages 50-69 years, higher household income, urban residence, pain in the teeth/mouth, impaired Oral Health Related Quality of Life, dental visit, having overweight or obesity and elevated total cholesterol were positively associated with poor SROH, and male sex, primary or less education and having 20 or more natural teeth were negatively associated with poor SROH. In addition, in the unadjusted analysis, having dentures, hypertension, diabetes, stroke, or heart attack were positively associated with SROH, and engaging in moderate or high physical activity were negatively associated with poor SROH.

**Conclusions:**

Almost one in ten participants reported poor SROH. Several factors associated with poor SROH were found that can aid in designing programmes to improve SROH in Sudan.

Oral diseases contribute to the large burden of public health problems worldwide, and poor oral health has significant detrimental effects on general health and quality of life.^35^ With the reorientation of health care towards becoming more patient-centerd, measuring the biopsychosocial dimensions of oral health, such as self-rated oral health (SROH), has recently become more relevant.^35^ Assessing determinants of SROH can help in understanding dental service utilisation and can improve dental health care.^34^ Studies have shown that SROH is significantly correlated with the individual’s clinical oral health.^6^ There are hardly any studies investigating the prevalence and correlates of SROH in population-based surveys among adults in African countries, including Sudan.^28,29,33^ Sudan is a low-income country and the prevalence and correlates of SROH may be different from high-income countries, considering that factors influencing SROH may be determined by country-specific sociocultural factors.^34,35^

In a survey-based study on the clinical oral health status of Sudanese adults in Khartoum State, a high prevalence of caries was found.^14^ Oral cancer has been reported as the 11th most common cancer in Sudan.^11^ In the 2015 national STEPS survey in Kenya, 13.7% of participants reported poor SROH,^33^ in South Africa 23.7% did so,^29^ in Nigeria 9%,^28^ in Australia 17.0%,^23^ in the USA 34.4%,^25^ and similar proportions of poor SROH have been reported in other countries as well.^8,31,36^

As previously reviewed,^33^ factors associated with poor SROH include sociodemographic factors (older age, female and low socioeconomic status), oral conditions (toothache, caries, sensitive teeth, bad breath, bleeding gums, and reduced number of teeth), general health status (overweight, obesity, and diabetes), oral health risk behaviour (inadequate toothbrushing, low oral hygiene behaviours, dental attendance), and general health risk behaviours (problem drinking, tobacco use, poor diet, avoidance of certain foods, and physical inactivity).

The aims of this study were to estimate the prevalence and correlates of SROH among adults in the 2016 Sudan STEPS survey.

## Materials and Methods

### Participants and Procedures

The sample included 7722 adults (18–69 years, median age 36 years; interquartile range: 23–43) who participated in the cross-sectional 2016 Sudan STEPS Survey.^11^ A nationally representative population-based sample was selected using a multistage sampling approach. The study response rate was 88%; further details and data are available elsewhere.^11^ Ethical approval was granted by the National Ethics Committee at the Federal Ministry of Health, Sudan, and participants provided verbal informed consent.^11^

### Study Measures

SROH was sourced from two items: 1- “How would you describe the state of your teeth,” and 2. “How would you describe the state of your gums?”^11^ Poor SROH was defined as having poor or very poor status of teeth and/or gums, and good oral health as having average, good, very good or excellent status of teeth and/or gums, in line with previous research.^33^ Cronbach’s alpha for the two-item SROH scale was 0.91 in this sample.

Oral health-related behaviours were sourced from three items: 1) “How often do you clean your teeth?” (1 = never, to 7 = twice or more a day); 2) “Do you use toothpaste?”(yes or no); 3) “How long has it been since you last saw a dentist?” (1 = less than 6 months ago, to 6 = never received dental care).^11^

Oral health-related quality of life (OHRQoL) was measured with ten questions, e.g. “Do you have difficulty in chewing foods?” (yes, no).^11^ Impaired OHRQoL was defined as a “yes” to any of the 10 OHRQoL questions. Cronbach’s alpha for the OHRQoL scale was 0.81 in this study.

Physical symptoms were sourced from the item “During the past 12 months, did your teeth or mouth cause any pain or discomfort?” (yes, no).^11^

Oral conditions included 1. tooth loss defined as having 0–19 teeth (vs having 20 or more teeth), and 2. dentures, “Do you have any removable dentures?” (yes, no).^11^

Inadequate fruit and vegetable intake was defined as less than five per day.^11^ Following the “Global Physical Activity Questionnaire (GPAQ)”, physical activity level was grouped into high, moderate, or low.^37^ Heavy episodic drinking was defined as having six or more standard alcoholic drinks on a single drinking occasion during any of the past 30 days.^11^

Using WHO STEPS standard methodology,^38^ hypertension (BP) was defined as systolic BP ≥ 140 mm Hg and/or diastolic BP ≥ 90 mm Hg or currently on antihypertensive medication (mean of the last two of three readings), and general overweight as Body Mass Index (BMI) 25–29.9 kg/m^2^ and obesity as BMI ≥ 30 kg/m^2^.^11^ Diabetes was defined as fasting plasma glucose levels ≥ 7.0 mmol/L, and/or currently taking insulin or oral hypoglycemic drugs,^11^ and elevated total cholesterol (TC) as fasting TC ≥ 5.0 mmol/L or currently on medication for elevated cholesterol. Blood glucose and cholesterol were measured using cardio-check examination equipment (Cardio check P.A., Polymer Technology Systems; Indianapolis, IN, USA).^11^

### Data Analysis

Statistical procedures were conducted with STATA v. 14.0 (Stata; College Station, TX, USA), taking into account the multistage sampling design and weighting of the data. Frequencies, percentages, means/medians, and standard deviations/interquartile ranges were used to describe the sample and health characteristics. Simple and multivariable logistic regression was utilised to estimate predictors of poor SROH. Variables statistically significant in the simple model were subsequently included in the multivariable logistic model. Missing values (< 5%) were excluded from the analysis, and p < 0.05 indicated significance.

## Results

### Characteristics of the Sample and Oral Health Indicators

In all, 7722 persons (median age 31 years, IQR: 23–43, range 18–69 years) were included in the study sample, 35.1% were males and 64.9% were females. The majority (62.9%) of the participants lived in rural locations, 51.5% had a household income ≤ 1000 Sudanese pounds, and 33.8% could not read and write. In relation to oral health behaviour, 63.2% of respondents cleaned their teeth ≥ 2 times/day, 87.5% used toothpaste, and 64.6% had never seen a dentist. Almost one in three (30.5%) of the participants had impaired OHRQoL, 1.3% had removable dentures, 10.8% had tooth loss (0–19 natural teeth), and 23.1% had experienced pain in the mouth or teeth in the preceding 12 months.

Regarding general health status, 28.3% of the sample had overweight/obesity, 1.2% had a history of stroke or heart attack, 5.9% had diabetes, 13.7% elevated total cholesterol, and 31.5% hypertension. In terms of general health behaviour, 15.6% of participants were current tobacco users, 1.8% had episodic heavy drinking in the past month, 94.7% consumed inadequate fruit and vegetables, and 78.8% had moderate or high physical activity. Almost one in ten participants (8.0%) reported poor SROH, 12.4% among females and 4.4% among men (Table 1).

**Table 1:** Sample and oral health characteristics, Sudan STEPS survey, 2016

Variables	Sample	Self-rated poor oral health
	N (%)	% (95% CI)
All	7722	8.0 (7.0, 9.1)
Age group (in years)		
18–34 35–49 50–69	3454 (57.7) 2474 (26.5) 1794 (15.8)	4.8 (3.8, 6.0) 9.8 (8.2, 11.5) 16.9 (14.4, 19.7)
Sex		
Female Male	5016 (64.9) 2707 (35.1)	12.4 (11.0, 13.9) 4.4 (3.4, 5.6)
Education		
Cannot write or read Primary or less More than primary	3272 (33.8) 2481 (34.2) 1952 (32.0)	10.6 (8.9, 12.5) 5.1 (4.2, 6.2) 8.4 (6.9, 10.4)
Household income		
≤500 ≤1000 ≤2000 >2000 Do not know	1326 (18.6) 2632 (32.9) 1949 (24.3) 727 (10.4) 1025 (13.7)	6.1 (4.6, 8.0) 6.7 (5.5, 8.1) 8.6 (7.2, 10.3) 14.1 (11.2, 17.5) 8.2 (5.2, 12.7)
Residence		
Rural Urban	5129 (62.9) 2593 (37.1)	6.5 (5.4, 7.7) 10.6 (8.9, 12.6)
Number of teeth		
0–19 20 or more	1031 (10.8) 6533 (89.2)	28.2 (23.6, 33.2) 5.8 (4.9, 6.8)
Dentures (removable)		
No Yes	7599 (98.7) 123 (1.3)	7.9 (6.9, 8.9) 20.1 (11.7, 32.2)
Pain in teeth/mouth (past year)		
No Yes	5662 (76.9) 2060 (23.1)	4.0 (3.4, 4.8) 21.1 (18.5, 24.0)
Impaired Oral Health Related Quality of Life		
No Yes	5088 (69.5) 2634 (30.5)	3.4 (2.7, 4.2) 18.5 (16.1, 21.0)
Teeth cleaning		
<twice/day ≥twice/day	2947 (36.8) 4775 (63.2)	8.7 (7.0, 10.6) 7.6 (6.6, 8.8)
Uses toothpaste		
No Yes	979 (12.5) 6731 (87.5)	7.0 (5.2, 9.4) 8.1 (7.1, 9.3)
Consultation with dentist		
Never >12 months ago Past 12 months	4716 (64.6) 1884 (22.0) 1122 (13.4)	4.0 (3.1, 5.1) 12.9 (11.0, 15.1) 19.1 (16.3, 22.4)
Current tobacco smoking	463 (9.0)	4.0 (2.7, 6.0)
Current smokeless tobacco use	458 (7.9)	7.9 (5.2, 11.8)
Heavy episodic drinking	78 (1.8)	5.3 (1.8, 14.3)
Inadequate fruit and vegetable intake	7220 (94.7)	8.0 (7.0, 9.1)
Physical activity		
Low Moderate High	1827 (21.2) 2023 (23.7) 3771 (55.1)	6.8 (8.5, 12.4) 8.0 (6.6, 9.5) 6.8 (5.6, 8.3)
Overweight/obesity	2455 (28.3)	10.8 (9.2, 12.6)
Hypertension	2710 (31.5)	11.0 (9.5, 12.6)
Elevated total cholesterol	1229 (13.7)	15.2 (12.4, 18.4)
Diabetes	515 (5.9)	15.1 (11.7, 19.4)
Stroke or heart attack	117 (1.2)	17.2 (10.8, 26.2)

### OHRQoL and SROH

Table 2 shows that the most common item of OHRQoL was “difficulty chewing” (17.5%), and that 30.5% had some impairment in OHRQoL. For all ten OHRQoL items, the prevalence of poor SROH was significantly (p < 0.001) higher than in persons with good SROH (see Table 2).

**Table 2:** Oral Health Related Quality of Life (OHRQoL) and self-rated oral health (SROH)

Variable	Overall	Poor SROH	Good SROH
OHRQoL items	N (%)	%	%
Difficulty in chewing	1594 (17.5)	24.1	4.6
Sleep often interrupted	1187 (13.3)	21.6	5.9
Feel tense	897 (10.2)	27.7	5.8
Doing usual activities	638 (7.0)	22.2	6.9
Days taken off work	469 (6.1)	17.6	7.4
Reduced social activities	495 (5.2)	20.3	7.3
Difficulty with speech	476 (5.1)	28.1	6.9
Less tolerant of spouse or someone close	411 (4.8)	22.1	7.3
Avoid smiling	391 (4.3)	25.1	7.2
Embarrassed about teeth	330 (3.5)	35.6	7.0
Overall OHRQoL			
Impaired OHRQoL	2634 (30.5)	18.5	3.4

### Associations Between Sociodemographic, Oral and General Health Variables with Poor SROH

In multivariable logistic regression analysis, ages 50–69 years, higher household income, urban residence, pain in the teeth/mouth, impaired OHRQoL, dental visit, having overweight or obesity and elevated total cholesterol were positively associated with poor SROH. Male sex, primary or less education and having 20 or more natural teeth were negatively associated with poor SROH. In addition, in the unadjusted analysis, having dentures, hypertension, diabetes, stroke, or heart attack were positively associated with SROH and engaging in moderate or high physical activity were negatively associated with poor SROH (see Table 3).

**Table 3:** Simple and multivariable logistic regression with poor self-rated oral health status

Variables	COR (95% CI)	p-value	AOR (95% CI)	p-value
Age group (in years)				
18-34	1 (Reference)		1 (Reference)	
35-49	2.16 (1.70, 2.75)	<0.001	1.18 (0.89, 1.56)	0.259
50-69	4.06 (3.03, 5.44)	<0.001	1.75 (1.21, 2.52)	0.003
Sex				
Female	1 (Reference)		1 (Reference)	
Male	0.32 (0.25, 0.42)	<0.001	0.49 (0.36, 0.66)	<0.001
Education				
Cannot write or read	1 (Reference)		1 (Reference)	
Primary or less	0.46 (0.35, 0.60)	<0.001	0.65 (0.48, 0.89)	0.007
More than primary	0.78 (0.59, 1.03)	0.077	0.79 (0.55, 1.13)	0.199
Household income				
≤500	1 (Reference)		1 (Reference)	
≤1000	1.11 (0.78, 1.57)	0.554	0.92 (0.61, 1.41)	0.713
≤2000	1.45 (1.04, 2.03)	0.027	1.23 (0.81, 1.88)	0.331
>2000	2.53 (1.70, 3.75)	<0.001	1.74 (1.02, 2.97)	0.043
Do not know	1.38 (0.79, 2.43)	0.260	1.50 (0.79, 2.85)	0.215
Geolocality				
Rural	1 (Reference)		1 (Reference)	
Urban	1.72 (1.31, 2.26)	<0.001	1.43 (1.06, 1.91)	0.022
Pain in teeth/mouth (past year) (yes)	6.38 (5.08, 8.01)	<0.001	2.39 (1.69, 3.39)	<0.001
Impaired Oral Health Related Quality of Life	6.47 (1.55, 5.62)	<0.001	2.74 (1.98, 3.79)	<0.001
Dentures (removable) (yes)	2.95 (1.55, 5.62)	<0.001	0.70 (0.31, 1.57)	0.388
Number of teeth (20 or more)	0.16 (0.12, 0.20)	<0.001	0.21 (0.15, 0.30)	<0.001
Teeth cleaning (≥twice/day)	0.87 (0.67, 1.13)	0.303	---	
Uses toothpaste	1.17 (0.85, 1.61)	0.334	---	
Consultation with a dentist				
Never	1 (Reference)		1 (Reference)	
>12 months ago	3.58 (2.65, 4.84)	<0.001	1.87 (1.32, 2.65)	<0.001
Past 12 months	5.72 (4.17, 7.85)	<0.001	1.83 (1.23, 2.71)	0.003
Current tobacco smoking	0.46 (0.30, 0.69)	<0.001	0.74 (0.41, 1.32)	0.304
Current smokeless tobacco use	0.99 (0.63, 1.54)	0.954	---	
Heavy episodic drinking	0.64 (0.21, 1.90)	0.420	---	
Insufficient fruit/vegetable consumption	0.97 (0.63, 1.49)	0.879	---	
Physical activity				
Low	1 (Reference)		1 (Reference)	
Moderate	0.75 (0.59, 0.96)	0.024	0.85 (0.63, 1.15)	0.289
High	0.63 (0.48, 0.83)	<0.001	1.05 (0.78, 1.41)	0.766
Overweight/obesity	1.57 (1.24, 1.98)	<0.001	0.76 (0.58, 0.99)	0.039
Hypertension	1.70 (1.40, 2.06)	<0.001	1.26 (0.98, 1.61)	0.070
Elevated total cholesterol	2.41 (1.89, 3.06)	<0.001	1.38 (1.04, 1.86)	0.032
Diabetes	2.18 (1.59, 2.99)	<0.001	1.14 (0.76, 1.70)	0.523
Stroke or heart attack	2.42 (1.37, 4.27)	0.002	1.39 (0.76, 2.55)	0.280

COR: Crude Odds Ratio; AOR: Adjusted Odds Ratio; CI: Confidence Interval.

## Discussion

The prevalence of poor SROH (8.0%) in this first national study in Sudan is similar to a study in Nigeria (9%),^28^ but lower than in Kenya (13.7%),^33^ Australia (17.0%),^23^ South Africa (23.7%),^29^ and the USA (34.4%).^25^ In line with previous clinical assessments of the oral health status among Sudanese adults in Khartoum State,^14^ this SROH study showed a high burden of oral health problems and great unmet treatment need.

In agreement with several previous studies,^8,23,26,29,33^ this study found that older age and female sex was associated with poorer SROH. Different explanations have been offered for the gender differences of the prevalence in SROH, including the fact that women more likely to report oral symptoms, are more self-critical, and have less access to oral health care than do men.^29^ Previous studies found an association between low socioeconomic status, urban residence,^7,8,18,19,23,29,36^ and the prevalence of poor SROH; similarly, in this study lower education was associated with poor SROH, but higher household income was positively associated with poor SROH. It is possible that lower general education translates into lower oral hygiene behaviour, an unhealthy diet and poorer access to oral health services, leading to poorer SROH and greater need for dental health care.

Consistent with previous studies,^3,13,16,22,30^ this study found in adjusted and unadjusted analysis that several oral problems, including impaired OHRQoL, pain in teeth/mouth, tooth loss, and dentures, were associated with poor SROH. As found previously in a study about chewing ability in Sudan,^15^ this survey showed a high correlation between difficulty in chewing/biting and poor SROH. Furthermore, in the adjusted and unadjusted analyses, various chronic conditions (stroke or heart attack, overweight/obesity, elevated total cholesterol, hypertension, and diabetes) were found to be associated with poor SROH. These findings are consistent with previous studies.^7-9,24,33^

Unlike some previous studies,^26,29^ this study did not show significant associations between oral hygiene behaviours (teeth cleaning and using toothpaste) and SROH. It is possible that due to the large proportion of inadequate tooth cleaning (36.8%), no such differences were found in terms of SROH. Regarding the association between dental attendance and poor SROH, previous research showed mixed results.^2,4,7,24,29,31,36^ This study found a positive association between dental attendance and poor SROH, which is consistent with some research studies.^2,4,7^ This finding may be attributed to the fact that 89.4% of the sample consulted a dentist because of pain or treatment (analysis not shown). Similar results were found in a study in Khartoum State, Sudan, where 91% visited the dentist because of pain.^14^

A number of studies^4,5,7,8,21,23,27,29^ showed an association between inadequate physical activity, insufficient fruit and vegetable intake, substance use (tobacco and alcohol) and poor SROH, but this study only found such an association in the unadjusted analysis in the case of physical inactivity. Nevertheless, previous research seems to indicate strong associations between tobacco use and poor dental health, as well as between smokeless tobacco use and oral cancer.^10,12,17^

### Study Strengths and Limitations

The strength of this study was the large nationally representative sample in Sudan, using the standard WHO STEPS survey methodology. The study was limited due to its cross-sectional design and self-reported oral health data. In addition, as Liu et al^19^ noted, patients are generally less likely to adequately assess their periodontal status and the presence of caries than they are to assess the number of their teeth, restorations, and the presence of fixed and removable prosthetics. Furthermore, some relevant variables, such as the intake of sugar-containing foods and soft drinks, were not available in the WHO Sudan 2016 STEPS survey dataset.

## Conclusion

Almost one in ten participants reported poor SROH and several associated factors were identified, including female sex, older age (50–69 years), no formal education, higher household income, urban residence, having less than 20 natural teeth, having pain in the mouth or teeth, impaired OHRQoL, dental visits, having overweight or obesity and elevated total cholesterol. This information can assist health care providers and policy makers in identifying priority groups in the Sudanese population for oral and dental health promotion activities. The delivery of oral health services should be strengthened throughout Sudan by focusing on older urban women, emphasising preventive dental care and integrating oral health into chronic disease management. In addition, community-based oral health promotion helps to empower communities to tackle oral health issues in their area.
